# Improving Robot Motor Learning with Negatively Valenced Reinforcement Signals

**DOI:** 10.3389/fnbot.2017.00010

**Published:** 2017-04-03

**Authors:** Nicolás Navarro-Guerrero, Robert J. Lowe, Stefan Wermter

**Affiliations:** ^1^Knowledge Technology, Informatics Department, University of Hamburg, Hamburg, Germany; ^2^Division of Cognition and Communication, Department of Applied IT, University of Gothenburg, Gothenburg, Sweden; ^3^Interaction Lab, School of Informatics, University of Skövde, Skövde, Sweden

**Keywords:** reinforcement learning, inverse kinematics, nociception, punishment, self-protective mechanisms

## Abstract

Both nociception and punishment signals have been used in robotics. However, the potential for using these negatively valenced types of reinforcement learning signals for robot learning has not been exploited in detail yet. Nociceptive signals are primarily used as triggers of preprogrammed action sequences. Punishment signals are typically disembodied, i.e., with no or little relation to the agent-intrinsic limitations, and they are often used to impose behavioral constraints. Here, we provide an alternative approach for nociceptive signals as drivers of learning rather than simple triggers of preprogrammed behavior. Explicitly, we use nociception to expand the state space while we use punishment as a negative reinforcement learning signal. We compare the performance—in terms of task error, the amount of perceived nociception, and length of learned action sequences—of different neural networks imbued with punishment-based reinforcement signals for inverse kinematic learning. We contrast the performance of a version of the neural network that receives nociceptive inputs to that without such a process. Furthermore, we provide evidence that nociception can improve learning—making the algorithm more robust against network initializations—as well as behavioral performance by reducing the task error, perceived nociception, and length of learned action sequences. Moreover, we provide evidence that punishment, at least as typically used within reinforcement learning applications, may be detrimental in all relevant metrics.

## 1 Introduction and Motivation

Pain and punishment are essential feedback mechanisms for informing self-preservative behaviors and both can heavily shape agent behavior. In robotics research, these types of feedback are often studied and applied in isolation. For instance, pain is often used as a trigger of protective reflexes with little or no adaptation, while punishment is often used within reinforcement learning setups to prohibit the carrying out of certain action sequences. In the following section, we will discuss in more detail both types of feedback and their use in robotics research, and later, we will introduce an alternative way of using pain feedback in robot learning.

### 1.1 The Role of Pain in Robotics

Although it can be argued that robots do not need pain or will likely never be able to experience pain, the mechanisms involved in pain management, e.g., physiological and autonomic responses, attention, and learning, may be modeled and taken as guidance to improve robot performance. Pain can even be considered a mechanism for developing safety features for human–robot interaction scenarios. It may be critical, for instance, to implement safe human–robot interaction and collaboration without protective barriers as collision detection via the use of nociceptive withdrawal reflexes (Kuehn and Haddadin, 2016). Chronic pain could also be used as a way to signal malfunction or damage to the enclosure of the robot that could be harmful to humans or lead to accidents, for instance, after a collision, the shell of the robot may be damaged and its surface may become rough or sharp.

Pain denotes a complex psychological and neurophysiological mechanism used to protect the body from injury (Westlund and Sluka, 2013). The experience of pain involves most of the central nervous system (CNS). It elicits immediate autonomic responses, attracts attention, and its effects can have long-lasting behavioral repercussions; evidence for the neural registration of pain are the reports of pain in the absence of any physiological cause such as chronic pain (Staahl and Drewes, 2004). More specifically, pain is defined by the International Association for the Study of Pain (IASP)^1^ as “an unpleasant sensory and emotional experience associated with actual or potential tissue damage, or described in terms of such damage” (Bonica, 1979, p. 250).

Under the definition of the IASP, pain is always subjective and defined in terms of experience. Nociceptors, on the other hand, are not considered as pain, per se. This is in line with the fact that the same noxious stimulus is perceived differently under different circumstances or different internal states such as anxiety and expectation (McGrath, 1994; Brooks and Tracey, 2005). Because pain is an emotional experience rather than an absolute measurable phenomenon, we prefer to differentiate it from nociception, which we define as a descriptor of actual or potential physical damage in robots.

The pain system is responsible for processing pain signals in humans and other mammals. It consists of specialized receptors called nociceptors, several nociceptive pathways, and brain structures responsible for processing and modulating diverse responses called *nocifensive behaviors* such as somatic and autonomic responses, endocrine changes, affective responses, and memory (Westlund and Willis, 2012).

The activation of nociceptors may trigger a number of *nocifensive* responses or behaviors that include somatic and autonomic reflexes, endocrine changes, motivational and affective responses, the formation of memories, complex conscious pain responses, among others (Westlund and Willis, 2012). Nocifensive and defensive behaviors are hierarchically organized in a series of nested and increasingly complex control loops, which involve at least the periaqueductal gray, hypothalamus, stria terminalis, amygdala, and ultimately the cortex (Canteras, 2002; Blessing and Benarroch, 2012).

Autonomic reflexes can be considered as part of the primary group of nocifensive responses, e.g., inflammation, activation of the immune system, endocrine changes, vocalizations, and motor reflexes. For instance, responses related to noxious cutaneous stimuli, called *nociceptive withdrawal reflexes* or *nociceptive flexor withdrawal reflexes*, are designed to prevent or reduce tissue damage by eliciting fast motor responses (Gebhart and Schmidt, 2013, p. 2226). Nociceptive withdrawal reflexes are primarily defined by cutaneous noxious stimuli and characterized by large receptive fields and actuation over all muscle groups of the body part affected (Strominger et al., 2012; Gebhart and Schmidt, 2013). On the other hand, autonomic reflexes associated with visceral systems are more general, e.g., changes in heart rate, blood pressure, and respiration, up to complex behavioral responses such as scratching. The particular type of responses associated with visceral noxious stimuli are also known as pseudo-affective responses because they resemble affective responses associated with painful stimuli, but they are not able to prevent damage or eliminate the threat (Gebhart and Schmidt, 2013, p. 2277).

Although autonomic reflexes are essential to any autonomous agent, they are not enough to cope with a highly dynamic environment, thus *nocifensive responses*, such as avoidance, motivated and affective behavior, memory formation, and learning, are also needed. These nocifensive responses are elicited by a sophisticated network of sensorimotor pathways. For example, nocifensive behaviors related to muscle and joint pain are characterized by a decrease of force and joint use, and a decrease of the mechanical withdrawal threshold (Gebhart and Schmidt, 2013, pp. 2284–2289). Adaptive nocifensive responses are critical to cope with the changes in the organism body due to aging, to lesions, to environmental constraints or to extraordinary events. For instance, changes in gait may be required to prevent further injury, or sometimes it may be required to experience pain to escape from life-threatening situations; how and when those changes in behavior happen cannot be predetermined and need to be designed in accordance with the problem at hand.

#### 1.1.1 Use of Nociception in Robotics

From the possible responses triggered by nociceptors or the pain system, only those related to autonomic reflexes have been typically applied to robot behavior. Autonomic reflexes are hard-wired responses elicited by painful stimuli designed to prevent or reduce damage.

For instance, Kawaji and colleagues (Akayama et al., 2006; Matsunaga et al., 2008, 2012) modeled pain perception produced by mechanical stimuli in humans. Specifically, they focus on touch, pressure, and brief impacts on the skin of human upper fingertips and arms. They considered how physiological aspects of the human skin, such as elasticity, sensitivity, and nociceptor distribution, influence pain perception in terms of intensity and duration. To model participants’ responses, Kawaji and colleagues (Akayama et al., 2006; Matsunaga et al., 2008, 2012) proposed a 2-DoF mass-spring-damper system model of mechanical pain. In this model, the pain intensity and the duration of the pain perception are expressed by the position of the outermost mass, which is proportional to the force of impact. The model can also be tuned to emulate fast and slow pain responses.

This model was applied to a simple nociceptive withdrawal reflex (Matsunaga et al., 2005), i.e., when the robot collides with an obstacle, it moves away from the obstacle and is programmed to avoid the coordinates of the collision until the pain dynamics modeled with the suggested 2-DoF mass-spring-damper system disappear.

More recently, Kuehn and Haddadin (2016) suggested a more elaborated set of nociceptive withdrawal reflexes based on the intensity of a mechanical stimulus applied to a BioTac^®^ sensor. Here, the nociceptive signal was used to trigger appropriate withdrawal reflexes, change joint stiffness and position, and limit movement for a predetermined time. The extent and intensity of these changes are directly dependent on the strength of the detected nociceptive stimulus, which Kuehn and Haddadin (2016) divided into four categories: none, light, moderate, and severe.

Although mainly preprogrammed, such a detailed model could also be applied as a feedback signal for learning and it can even be speculated that such models may contribute to the development of some sort of empathic robots, i.e., robots that could judge subjective pain or tissue damage based on the observed speed, shape of the objects involved, among other variables.

Along with a similar line or research, however, instead of reacting to nociceptive signals other research groups have focused on preprogramming reflexes for anticipating pain, particularly to prevent a fall or reduce the damage from an imminent fall. Reactions to a lack of stability are meant to prevent a fall or to protect vital organs in an imminent fall. Due to the inherent instability of humanoid robots, it seems reasonable to imbue them with fast and adaptable collision and fall management systems instead of just reducing operational speed or posing restrictions on the robot’s workspace.

Shimizu et al. (2011, 2012) designed a reflex management architecture for the humanoid robot iCub. The architecture efficiently orchestrates preprogrammed responses against collisions and falls. The system was optimized for falls that start from a still and upright pose. Robot responses are divided into global and local reactions. Those reactions are meant to protect the robot’s head and torso while reducing the overall damage. Global reactions were designed to provide a whole body reaction when falling and local reactions were designed to respond to particular conditions while performing a global reaction sequence.

Another sophisticated example of a fall management system for robots was developed by Ruiz-del Solar et al. (2009, 2010). Similar to the system developed by Shimizu et al. (2012), this system focuses on the falls produced by external events and not inherent in the robot’s locomotion. It employs whole body reactions and the system can naturally cope with a fall from any robot position. But contrary to the system of Shimizu et al. (2012), here, the instabilities are evaluated in real time and thus can be used regardless of the robot’s gait or pose. The main drawback of the system is that it was optimized for robot soccer applications and thus suitable only on even surfaces.

Although of significant importance, these applications do not fully exploit the potential of nociceptive signals, as they are only used as triggers for preprogrammed behaviors. However, these last examples of reacting in anticipation of pain can be further developed using, for instance, reinforcement learning. Reinforcement learning can be used to improve upon preprogrammed behaviors or to learn new behaviors from scratch using nociceptive signals as guidance. However, nociceptive signals in reinforcement learning are rarely used in an embodied context as will be discussed in the next section.

### 1.2 Reinforcement Learning and Punishment

Reinforcement learning and particularly temporal-difference (TD) learning are the preferred algorithms to model learning by feedback, whether this is in the form of reward or punishment. Reinforcement learning algorithms follow a trial-and-error learning paradigm and are formalized according to the idea of an agent who learns from its experience, and whose task is to maximize the cumulative reward in the long term (Sutton and Barto, 1998, p. 56). However, no special treatment is given to punishment, which is simply modeled as a negative reward without further implications (Seymour et al., 2005, 2015; Balkenius and Winberg, 2008; Lowe and Ziemke, 2013; Palminteri and Pessiglione, 2017). Yet, recent evidence indicates that this may not be the case and that in fact punishment is processed by a different neural pathway than reward, at least for procedural or skill motor learning (Galea et al., 2015; Palminteri and Pessiglione, 2017). Moreover, punishment-driven learning may be a more demanding cognitive task than reward-driven learning (Wächter et al., 2009; Kim et al., 2015; Palminteri and Pessiglione, 2017).

The use of punishment in applications of TD-learning algorithms is widespread and often used: (1) with the intention to limit the time spent in certain states or to avoid them altogether (Weber et al., 2004; Balkenius and Winberg, 2008) and (2) with the expectation to obtain solutions with shorter action sequences (van der Wal, 2012). The punishment signal is usually a numerical value devoid of any relationship to embodied perception and of which its effect on learning in general is typically not quantified. Moreover, most approaches do not take into account that punishment-driven learning may not be appropriately modeled by TD-learning algorithms and perhaps reinforcement learning algorithms in general (Palminteri and Pessiglione, 2017).

A wealth of research has identified the key brain regions involved in different aspects of reward- and punishment-driven learning, including the midbrain, the striatum, the amygdala, the orbitofrontal cortex, and the medial prefrontal cortex. Most findings shed light on the neural pathways involved in reward-seeking behaviors only; however, less is known about punishment-driven learning (Wächter et al., 2009; Kim et al., 2015) and the combined effects of both types of reinforcement on behavior learning (Dayan and Niv, 2008; Wächter et al., 2009). Evidence suggests that there are substantial neurobiological differences (Wächter et al., 2009; Kim et al., 2015). For instance, the striatum, the amygdala, and the medial OFC seem to be more involved in reward-driven learning, while for punishment-driven learning, the insula or the lateral OFC play a greater role. Moreover, Wächter et al. (2009) and Kim et al. (2015) show the differential involvement of the striatum in reinforcement learning tasks that require action execution. Specifically, the ventral striatum has been linked to reward anticipation, while the dorsal striatum has been associated with both reward and punishment anticipation and thus valence-free action value representations. These findings support existing evidence that the ventral striatum is involved in reward-driven learning, whereas the dorsal striatum is associated with the formation of habits during reward learning (Kim et al., 2015).

During feedback, punishment-driven rather than reward-driven learning elicits a greater engagement of the prefrontal cortex and thus more attentional and cognitive resources are recruited (Kim et al., 2015). This could indicate that punishment-driven learning is a more complex and cognitively demanding process. Dayan and Niv (2008) agreed with this view and argue that this may be due to the heterogeneous range of effects that aversive predictions elicit, which greatly depend on contextual information.

A behavioral study on a procedural learning task by Wächter et al. (2009) found that reward-driven learning can lead to significantly higher performance than punishment-driven learning, where performance is measured with respect to reaction time and error rate. By contrast, punishment did not have an effect on learning, but did have an effect on behavioral aspects, e.g., leading to an immediate reduction of the reaction time, when tested on a sequence-less version of the task (Wächter et al., 2009).

These results could be considered contradictory to other studies (Hester et al., 2010), where punishment-driven learning has been found to improve learning performance; however, the nature of the tested tasks is different, i.e., procedural learning vs. associative learning, respectively. Besides, the recognized existence of differential pathways for reward- and punishment-driven learning reconciles both results.

#### 1.2.1 Use of Punishment in Robotics

Attempts to fill the knowledge gap of TD-learning models with respect to punishment-driven learning and its effects when combined with reward are still scarce. In a rare example, Tamosiunaite et al. (2009) studied the effect of different reward and punishment strategies in a learning-to-reach task. They used 4 types of strategies all based on reward only but the best strategy was further studied in combination with punishment. The first strategy was *reward only*, the reward here was only given once the target had been reached. The second strategy was based on the *distance to the target*, i.e., the closer to the target the greater the reward. The third strategy combined both aforementioned strategies and was tested using two variants. Both variants gave a *distance-based reward* and a bonus once the target was reached, but one of the variants gave the distance-based reward only if the distance was minimized (*approach-distance-based* reward). Finally, the fourth strategy was *differential*, here the agent was given a reward based on how much the distance to the target was minimized. From the tested conditions, the *approach-distance-based* reward plus bonus-on-touch converged faster and required shorter action sequences than the other strategies.

Further, the *approach-distance-based* reward in combination with punishment on joint constraint was analysed. Here, a punishment is given when the joint reaches either limit. This new strategy increased convergence speed and worked better over a larger range of hyperparameters. Tamosiunaite et al. (2009) also tested *punishment as a constraint* together with *punishment when moving away from the target* but this had no impact on their results.

Balkenius and Winberg (2008) studied a modular Actor-Critic architecture with an additional module (*Punish*) that learns state-action pairs that lead to punishing states in maze-navigation tasks. All three modules, i.e., the *Actor, Critic*, and *Punish*, encode the current state differently. The *Actor* learns only using local information as input. Specifically, it uses a matrix of binary values indicating whether obstacles are present in the current state or immediately neighboring states. This facilitates generalization while also informs the *Actor* of possible actions in each state narrowing the search space. The *Critic* uses global position information for learning. This seems more appropriate for evaluating the actions chosen by the *Actor* in a goal-directed manner by creating a gradient toward the goal. Finally, the *Punish* module encodes obstacle configuration around the agent into mutually exclusive classes. These classes encode specific state-action pairs that are not generalized to achieve *context-specific* responses. In this way, the *Punish* module can further reduce the search space of the *Actor* when in a given state by actively suppressing actions that lead to known aversive outcomes, in other words, it tells the *Actor* what actions to avoid when in a given state. Balkenius and Winberg (2008) showed that having dedicated modules for aversive and appetitive learning improved learning speed by reducing the total number of steps required to solve a maze.

A more neurally motivated study was presented by Lowe and Ziemke (2013). Here, rather than focusing on the task or the embodied nature of the nociceptive signals, Lowe and Ziemke (2013) tested alternative ways of representing reward and punishment, and how these representations are later combined for decision-making in an n-armed bandit navigation task. These representations are updated independently and combined into an action value function (*Q_rp_*) used for action selection. Here, the value representation of reward (*Q_r_*) is linearly modulated by the value representation of punishment (*Q_p_*), so that *Q_r_* is inhibited as *Q_p_* increase. Additionally, two meta parameters are used to influence the probability of exploration. Similarly, as for the action value function, an internal representation of reward R is linearly modulated by an internal representation of punishment P, where strong punishment inhibits behaviors associated with reward. This method encompasses many reinforcement contingencies modulated by the expected reward and punishment that are observable in context-dependent levels of exploration versus exploitation.

The abovementioned evidence presented in Section 1.1 and Section 1.2 motivates this research. Here, we study to what extent reward in combination with punishment and nociceptive input affects agent behavioral performance and motor skill learning capabilities during an inverse kinematics learning scenario. We chose a version of the well-established TD-learning algorithm to evaluate their suitability for capturing the differential dynamics of reward- and punishment-driven learning. In contrast to the existing work on computational nociceptive signals and punishment where they are either used as a trigger of reactive or anticipatory preprogrammed behavior or have little or no embodied origin, here we suggest an embodied approach where nociceptive information is used to aid learning by increasing the state space or as a negative reward. In this way, we improved learning performance (indicated as the number of learning steps needed) and behavioral performance (measured as cumulative positioning error) on an inverse kinematic learning task.

## 2 Task Description and Methodology

Despite advances in humanoid robot control, there are still challenges regarding generalization and autonomous learning of new tasks. One of these tasks is robust object reaching. Although this task is actively being studied, e.g., Tamosiunaite et al. (2009), van der Wal (2012), and Stahlhut et al. (2015), due to its number of applications for industrial and domestic robots, it is still challenging and many aspects remain to be studied such as self-calibration, adaptation, learning of speed, and force control.

Evidence from both child development research (Thelen et al., 1993) and adult novel sensorimotor task learning (Franklin et al., 2007) suggests that learning to reach does not require visual feedback, but seems to be useful for fine correction at the end of a reaching movement. Moreover, in early infancy, motor programs for reaching are not explicitly planned ahead of a movement (trajectory planning), which points at a trial-and-error learning paradigm. Reinforcement learning methods are particularly suitable for this type of learning.

Actor-Critic architectures are powerful TD-learning methods that model phasic changes in dopamine neuron activity (Suri, 2002). The Critic guides the learning of action sequences generated by the Actor in order to maximize the accumulated reward. The dual memory structure, one for the Critic and one for the Actor, allows storing the learned policy explicitly, which significantly reduce the computation of action selection of large state and action spaces when compared to other TD learning methods (Sutton and Barto, 1998, p. 153). Moreover, Actor-Critic methods are thought to be consistent with biological evidence (Suri, 2002). This is due to the fact that the reward prediction signal of TD learning resembles the dopamine neuron activity in the striatum. Also, the Actor typically connects a high-dimensional sensory input to a smaller action space, which resembles its neural equivalent, i.e., projections from the striatum to the basal ganglia output nuclei (Suri, 2002).

### 2.1 Experimental Setup

Here, the problem of autonomous learning or inverse kinematics of a single robot arm is addressed. The robot’s objective is to move the geometrical center of its end-effector toward a target as precisely and as quickly as possible. Arm movements are controlled using motor commands relative to the current joint position, but no inverse or direct model of the arm dynamics is provided to the agent.

Because our main interest lies in the effects of punishment and nociception on the learning of motor skills, a number of simplifications are made. A simplified 2-degrees-of-freedom model of a NAO robot arm is used, i.e., restricted to only one shoulder and one elbow joint. The link lengths are 105 mm for the upper arm, and 113.7 mm in total for the lower arm and hand.^2^ The shoulder joint is limited to the range [−18, 76] degrees and the elbow joint is limited to the range [−88.5, −2] degrees.^3^ The robot is able to precisely perceive the target’s position in an egocentric reference frame, i.e., exteroception. It can also precisely perceive the absolute angular position of its joints, i.e., proprioception. It can perceive when the joints are at or close to their upper or lower limits, i.e., interoception (nociception). Nociceptive input is maximal when a joint is at the mechanical limit and decreases exponentially as the joint moves away from the limit. Nociception is perceived only when the current joint position is within the upper or the lower 10% of its mechanical range. Reaching is considered successful when the robot’s hand is at most 10 mm away from the target.

**Figure 1:**
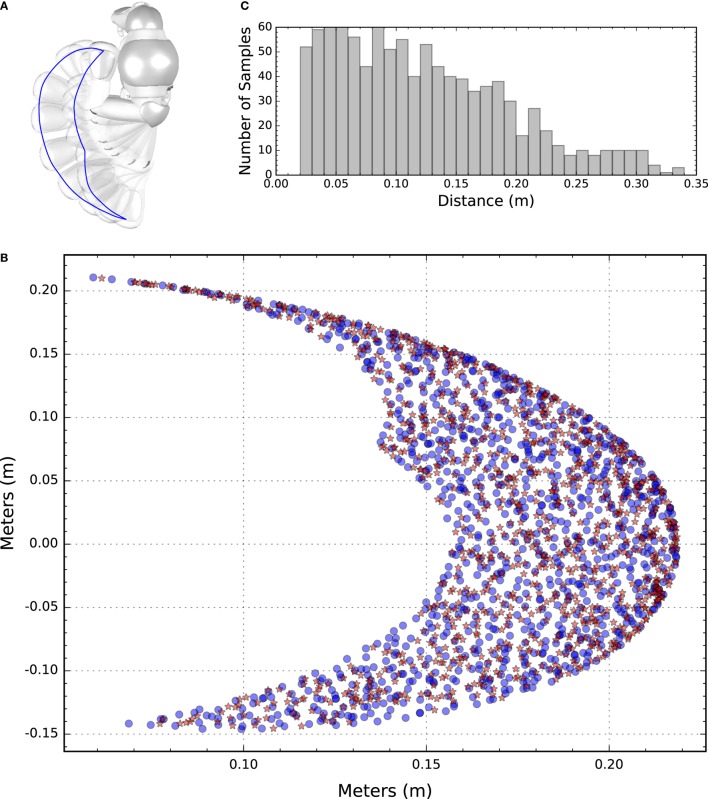
**(A) Top view of the NAO robot facing left**. The left arm is depicted in different positions and a blue line is superimposed to indicate the boundaries of the end-effector workspace. **(B)** Depiction of the target and end-effector coordinates of the randomly generated training set. Blue dots represent targets, whereas red asterisks represent end-effector initial positions. **(C)** The histograms show the initial distance between the end-effector and the corresponding target.

To compare different learning conditions a unique training, test and validation set for all conditions was used of sample size 1,000, 100, and 1,000, respectively. Each sample consists of a target in Cartesian coordinates and an initial joints configuration in degrees (see Figure 1). Samples are randomly generated and the resulting end-effector positions are at least twice the reaching threshold of 10 mm apart. Training, test, and validation samples are always presented in the same order. A complete presentation of the training set is termed “epoch.” Before any learning is performed, the agent is tested on the test set, and after each epoch afterward. The test set is used to determine a winning set of hyperparameters for all conditions used, whereas the validation set is used for a detailed comparison between conditions.

We compared four learning conditions. The first condition, *reward only (R)*, and our baseline consisted of an agent trained using the target and current joint position information as state space, and it received only a binary reward once the desired goal state was reached. The second condition, *reward* + *punishment (R* + *P)*, used the same state space of the *reward only* condition but extended the binary reward by incorporating a punishment term directly derived from the perceived nociception. The third condition, *reward* + *nociception (R* + *N)*, used the same binary reward as the *reward only* condition but extended the state space by including one nociceptive unit per joint. Finally, the fourth condition, *reward* + *punishment* + *nociception (R + P + N)*, used the state space of the *reward* + *nociception* condition and the reward and punishment of the *reward* + *punishment* condition.

### 2.2 Continuous Actor-Critic Learning Automaton (CACLA)

CACLA (van Hasselt and Wiering, 2007) is a model-free reinforcement learning algorithm with Actor-Critic architecture. This algorithm was designed to work with large and continuous state and action space, thus an excellent alternative to learn the problem described in Section 2.1. These characteristics are obtained through the use of function approximation techniques such as feed-forward multilayer perceptron neural networks (MLP) that allow, for example, generalization, see van der Wal (2012).

Actor-Critic methods are on-policy temporal-difference (TD)-learning methods that have two memory structures, i.e., a dedicated memory for policies and another for value functions. The Actor represents the policy and this is denoted as *A*(*s*). The Critic provides a state-value function *V* (*s*). The Critic evaluates the outcome of the selected action against its existing value estimate (expectation) and generates a TD error to the extent that it differs, see equation (1). The TD error is then used to update both the Actor and the Critic. If the error is positive, the selected action should be strengthened, whereas a negative error indicates the opposite (Sutton and Barto, 1998, p. 152). The TD error is defined as:

(1) $$\delta_{t}=r_{t}+\gamma V\left(s_{t+1}\right)-V\left(s_{t}\right)$$

where *r_t_* is the reward received at time *t*, γ is the discount factor of future rewards, *V* (*s_t_*_+1_) is the expected reward at the state *s_t_*_+1_, and *V* (*s_t_*) is the expected reward for state *s_t_*.

**Figure 2:**
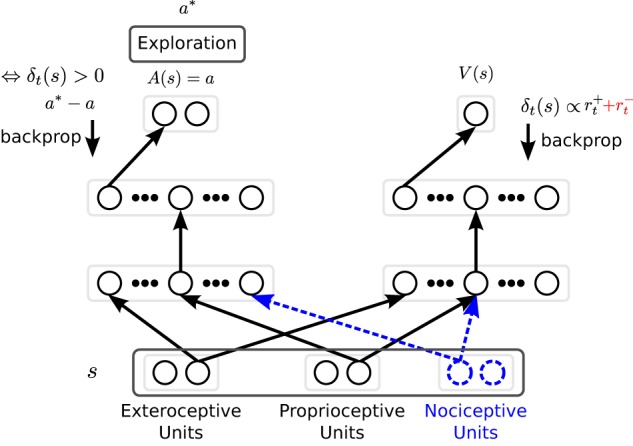
**The neural architecture used for inverse kinematics learning**. For clarity, only one connection weight is shown (arrow between neuron layers). The hidden layers for both the Actor (left-hand side) and the Critic (right-hand side) are independently tuned. Solid units and connection weights in black correspond to the baseline, i.e., the *reward only* condition, and are extended by the other 3 conditions. The punishment feedback given to the critic and depicted in red is only used for the *reward* + *punishment*, and *reward* + *punishment* + *nociception* conditions. Blue dashed units and blue dashed connection weights are only considered under the *reward* + *nociception*, and *reward* + *punishment* + *nociception* conditions. During training a* is performed. a* is determined based on the exploration of action *a* as described in equation (2). The Critic is trained every time step based on the TD error δ, while the actor is trained only if the TD error is positive.

Action selection is based on the current policy but in order to discover new and better policies, i.e., to learn, exploration is required. We use Gaussian exploration, where the performed action is sampled from a Gaussian distribution centered at the Actor’s output *A*(*s_t_*). So the probability of selecting action *a* at time *t* is:

(2) $$p_{t}\left(s_{t},a\right)=\frac{1}{\sqrt{2\pi \sigma }}e^{-{\left(a-A\left(s_{t}\right)\right)}^{2}∕2\sigma^{2}}$$

where π denotes the mathematical constant and σ denotes the SD and is here also called exploration rate. Finally, the performed action determined by equation (2) and called a*, see Figure 2 for a graphical representation of our implementation of CACLA.

CACLA differs from conventional Actor-Critic systems (Sutton and Barto, 1998, p. 152) in that the magnitude of the Actor’s update is independent of the size of the TD error. The Actor is instead updated toward the explored action only when the sign of the TD error is positive. This idea originates from the fact that punishing or moving away from an action that does not lead to a higher reward does not guarantee a better solution (van Hasselt and Wiering, 2007). Thus, the Actor is only updated toward actions that improve agent performance instead of pulling the weights around without a destination. To control how strongly actions will be reinforced, a derived algorithm called CACLA + var is used (van Hasselt and Wiering, 2007). CACLA + var keeps a running average of the TD error’s variance, so actions leading to unusually higher rewards are reinforced proportionally higher:

(3a) $${\mathrm{var}}_{t+1}=\left(1-\beta \right)\mathrm{va}r_{t}+\beta \delta_{t}^{2}$$

(3b) $$\text{number}\;\text{of}\;\text{updates}=\left\lceil \delta_{t}∕\sqrt{\mathrm{va}r_{t}}\right\rceil$$

CACLA + var requires two additional parameters to be tuned, i.e., *var*_0_, which should be comparable to the typical value of δ, this is important to avoid high reinforcement rates early in learning when the agent behaviors are mostly random, and β.

Then, the Actor’s policy update can be expressed in pseudo-code as:

**Algorithm 1:** **Actor’s update**.

1: **if δ***_t_* > 0 **then**
2: **for** i: = 1 **to** $\left\lceil \delta_{t}∕\sqrt{\mathrm{va}r_{t}}\right\rceil$ **step** 1 **do**
3: $\theta_{i,t+1}^{A}=\theta_{i,t}^{A}+\alpha \left(a_{t}^{*}-A\left(s_{t}\right)\right)\;\frac{\mathrm{\partial A}\left(s_{t}\right)}{\partial \theta_{i,t}^{A}}$
4: **end for**
5: **end if**

where $\theta_{i,t}^{A}$ is the *i*th item of the parameter vector of the Actor at time *t, s_t_* is the state vector at time *t* and α is the learning rate for the Actor’s function approximator. Unlike the Actor, the Critic is updated every time step as follows:

(4) $$\theta_{i,t+1}^{V}=\theta_{i,t}^{V}+\eta \delta_{t}\frac{\mathrm{\partial V}\left(s_{t}\right)}{\partial \theta_{i,t}^{V}}$$

where $\theta_{i,t}^{V}$ is the *i*th item of the parameter vector of the Critic at time *t*, and η is the learning rate for the Critic’s function approximator.

### 2.3 Reward Function

The reward function consists of two parts, i.e., a rewarding component depending on the end-effector position and a punishing component depending on the joints’ position, which are additively combined into a single scalar value after every step. The rewarding component is computed as follows:

(5) $$r_{t}^{+}=\left\{\begin{array}{cc} R\; & \;,\;\text{if}\;d_{t}\leq 1.00\;\text{cm}\\ 0\; & \;,\;\text{otherwise} \end{array}\right.$$

where *R* is the highest reward value, and *d_t_* the distance from the end-effector to the target at time *t*.

Joint positions close to the lower or upper limit are considered harmful and a punishment signal is used to signal this. The amount each joint contributes to the total punishment per time step is computed as follows:

(6) $$r_{t}^{-}=-\frac{P}{\mathrm{dof}}\times \left\{\begin{array}{cc} 0\; & \;\;\;\;\;\;\;\;\;,\;\text{if}\;J_{i}^{\min}+m_{i}<j_{i}<J_{i}^{\max}-m_{i}\\ e^{-0.5{\left(\frac{j_{i}-J_{i}^{\min}}{m_{i}}\right)}^{2}}\; & \;\;\;\;\;\;\;\;\;,\;\text{if}\;J_{i}^{\min}+m_{i}\geq j_{i}\\ e^{-0.5{\left(\frac{j_{i}-J_{i}^{\max}}{m_{i}}\right)}^{2}}\; & \;\;\;\;\;\;\;\;\;,\;\text{if}\;j_{i}\geq J_{i}^{\max}-m_{i}\\ \; \end{array}\right.$$

where *P* is the maximum magnitude of punishment, *dof* the total number of degrees of freedom, *j_i_* the absolute angular position of the *i*th joint at time *t*. $J_{i}^{\min}$ and $J_{i}^{\max}$ are the minimum and the maximum possible angular position of the *i*th joint and *m_i_* is the margin of safety for a safety factor of 0.1 for the *i*th joint.

### 2.4 Neural Architecture

We use two MLPs, one for the Actor and one for the Critic (see Figure 2). Both share the same input layer. The output layer for the Actor has two units, one per degree of freedom of the robot arm. The Critic has a single output unit. The rest of the layout is determined separately. The input layer is divided into three perceptual modalities. First, there are two exteroceptive units which encode the Cartesian coordinates of the target in a 2-dimensional task space relative to the robot. Second, there are two proprioceptive units that encode the angular position of each of the joints of the robot’s arm, i.e., the absolute joint value of the shoulder and elbow joint. Finally, there are two nociceptive units associated with each robot joint, with an activation almost identical to the function of punishment, see equation (6). However, here nociceptive signals triggered by movements toward the lower limit of a joint are negative and positive for movements toward the upper limit.

All input values are scaled to the range [−1, 1]. The squashing function for the output units is linear, and for all other units, a custom hyperbolic tangent as defined by LeCun et al. (1998, 2012) is used. Weight initialization is also performed as defined by LeCun et al. (1998, 2012). Bias units with value −1 are always used. Momentum and learning rate decay are not used. Both networks, the one for the Actor and the one for the Critic, are trained using back-propagation.

The activation of each nociceptive unit, see equation (7), is computed in a similar way as the punishment signal, see equation (6). The only two differences are that nociceptive units are able to discriminate between the upper and lower range of each joint, and the magnitude of the activation of each unit is not limited by the maximum punishment value and is not affected by the number of degrees of freedom of the arm. We chose an exponentially decaying function to model nociception and punishment as a simplification of the model for phasic mechanical cutaneous pain suggested by Kawaji and colleagues (Akayama et al., 2006; Matsunaga et al., 2008, 2012).

(7) $$n_{t}=\left\{\begin{array}{cc} 0\; & \;,\;\text{if}\;J_{i}^{\min}+m_{i}<j_{i}<J_{i}^{\max}-m_{i}\\ -e^{-0.5{\left(\frac{j_{i}-J_{i}^{\min}}{m_{i}}\right)}^{2}}\; & \;,\;\text{if}\;J_{i}^{\min}+m_{i}\geq j_{i}\\ e^{-0.5{\left(\frac{j_{i}-J_{i}^{\max}}{m_{i}}\right)}^{2}}\; & \;,\;\text{if}\;j_{i}\geq J_{i}^{\max}-m_{i}\\ \; \end{array}\right.$$

### 2.5 Hyperparameter Optimization

Due to a large number of possible combinations of hyperparameters, the systematic and exhaustive testing of them is impractical. Thus, we decided to use a genetic algorithm (GA) to explore the hyperparameter space, which helps to discover novel solutions and to determine which hyperparameters have the greatest influence on performance. The hyperparameters to be optimized by the genetic algorithm along with the search space for each of them are detailed in Table 1. Because small changes in the hyperparameters are likely to produce little change in performance, we decided to discretize their values and thus the search space as indicated in Table 1.

**Table 1:** **List of hyperparameters for CACLA and MLP subject to evolutionary search**.

Parameter name	Symbol	Search space
CACLA + var beta	β	{*k* : *k* + 0.00025, 0.0001 ≤ *k* ≤ 0.0251}
Initial variance	*var*_0_	{*k* : *k* + 0.1, 2.0 ≤*k* ≤7.5}
Initial iterations	$\left\lceil \delta_{0}∕\sqrt{\mathrm{va}r_{0}}\right\rceil$	{*k* : *k* + 1, 5 ≤ *k* ≤15}
Discount factor	γ	{*k* : *k* + 0.001, 0.60 ≤ *k* ≤1.0}
Exploration rate	σ	{*k* : *k* + 0.1, 0.7 ≤ *k* ≤1.6}
Exploration rate decay	κ	{*k* : *k* + 0.001, 0.940 ≤ *k* ≤1.0}
Learning rate Critic	η	{*k* : *k* + 0.0025, 0.0001 ≤ *k* ≤0.175}
Critic MLP in →h1st	*C*_*in*→*h1st*_	{*k* : *k* + 5, 10 ≤ *k* ≤50}
Critic MLP h2nd →out	*C*_*h2nd*→*out*_	{*k* : *k* + 5, 0 ≤ *k* ≤25}
Learning rate Actor	α	{*k* : *k* + 0.0025, 0.0001 ≤ *k* ≤0.175}
Actor MLP in →h1st	*A*_*in*→*h1st*_	{*k* : *k* + 5, 10 ≤ *k* ≤50}
Actor MLP h2nd →out	*A*_*h2nd*→*out*_	{*k* : *k* + 5, 0 ≤ *k* ≤25}
Reward	*R*	{1, 10, 100}
Punishment	*P*	{−1, −0.1, 0}

*Variables range based on Navarro-Guerrero (2016)*.

Regarding the GA, a small randomly initialized population is used due to the computational cost of large populations. For practical reasons, we chose 32 individuals per generation, which corresponded to the number of cores we had available for parallel computation. The small number of individuals reduce the exploration in the initial generation, but this can be overcome with the use of evolutionary operators such as crossover and mutation (Schaffer et al., 1989). Elitism is used to preserve the best four solutions. The remaining 28 individuals are selected using *Tournament selection*, recombined using a single-point crossover, and finally mutated. Tournament selection is a simple selection method with an adaptable selection pressure, i.e., low when fitness distribution is high and vice versa, which also helps prevent premature convergence (Mitchell, 1998). Single-point crossover is also chosen due to its simplicity and efficacy with short genome encoding (Mitchell, 1998). A normally distributed mutation was used to explore the neighborhood of tested solutions but also allowed a certain degree of exploration. Each gene is mutated with a 10% probability and a sigma of 6.25% of the corresponding hyperparameter range, both percentage values were manually tuned. Finally, to foster exploration and reduce the likelihood of premature convergence, we only allow for testing on new genotypes, thus when an already tested genotype is produced, it is randomly mutated until an untested genotype is found.

The fitness function for the GA consists of the total distance between the robot’s end-effector and target on the testing set after learning, i.e., after the last epoch. Thus, here, the lower the fitness values the better. Equation (8) shows the mathematical formulation of the fitness function:

(8) $$D=\sum_{i=1}^{p}\;d\left(h_{i},t_{i}\right)$$

where *p* represents the total number of testing pairs, *h_i_* corresponds to the initial joint positions of the arm for testing pair *i, t_i_* corresponds to the coordinates of the target for testing pair *i*, and *d* is the final Euclidean distance between the arm’s end-effector and the corresponding target.

## 3 Experimental Results

**Figure 3:**
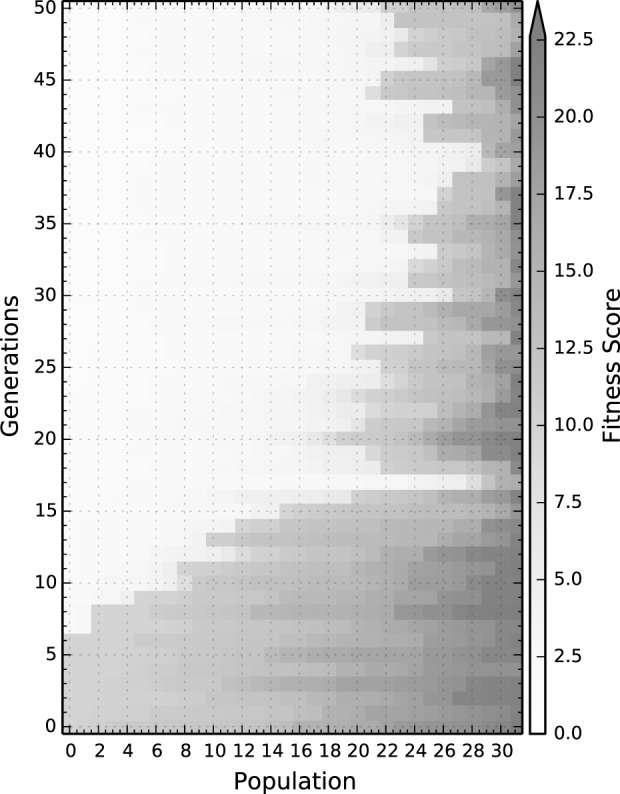
**A representative example of all four conditions of the fitness distribution over generations;** the results for each particular condition can be found in the Supplementary Material Navarro-Guerrero et al., 2017. The fitness is directly computed from the total distance to the target on the testing set once learning has been concluded, thus the lower the value the better.

Figure 3 shows an example of fitness values over time for the four conditions tested in this work, i.e.:

- agent trained with *reward only (R)*,
- agent trained with *reward* + *punishment (R* + *P)*,
- agent trained with *reward* + *nociception (R* + *N)*, and
- agent trained with *reward + punishment + nociception (R + P + N)*.

The population per condition and per generation is 32 individuals. Evolution is carried out for 50 generations after which convergence was observed for all tested conditions. The following subsections show the results for the best solution to each condition, which are validated by training them 10 times with different initializations. The reported behavioral results correspond to the average behavior of the 10 different initializations after learning and are tested on the validation set. The raw data can be downloaded from a dedicated page for Supplementary Material (Navarro-Guerrero et al., 2017).

At a macroscopic level, i.e., at the evolutionary level, all solutions are comparable both in terms of convergence speed as well as in the quality of the best solution. Nevertheless, there are differences in terms of the mean fitness, see Table 2. Particularly, solutions for conditions trained with punishment, when compared to conditions trained without punishment, have worse fitness values. This could indicate that the conditions with punishment are more difficult to learn and thus require more finely tuned hyperparameters.

**Table 2:** **Summary of best hyperparameters and fitness values at generation number 50**.

Parameter name	Symbol	*R*	*R* *+* *P*	*R* *+* *N*	*R* *+* *P* *+* *N*
CACLA + var beta	β	0.0251*	0.01685	0.0001*	0.0156
Initial variance	*var*_0_	5.1	4.4	4.7	4.3
Initial iterations	$\left\lceil \delta_{0}∕\sqrt{\mathrm{va}r_{0}}\right\rceil$	10	10	11	10
Discount factor	γ	0.836	0.887	0.715	0.868
Exploration rate	σ	1.4	1.2	1.3	0.9
Exploration rate decay	κ	1	1	1	1
Learning rate Critic	η	0.0001*	0.0276	0.0601	0.0076
Critic MLP in →h1st	*C*_*in*→*h1st*_	35	35	25	30
Critic MLP hsecond →out	*C*_*h2nd*→*out*_	10	15	10	15
Learning rate Actor	α	0.0426	0.1151	0.0226	0.0751
Actor MLP in →h1st	*A*_*in*→*h1st*_	30	30	30	30
Actor MLP hsecond →out	*A*_*h2nd*→*out*_	15	15	10	10
Reward	*R*	10	10	10	10
Punishment	*P*	N.A.	−0.1	N.A.	−0.1
Avg. fitness (SD)		6.253625 (±5.480689)	8.939111 (±6.389975)	4.765209 (±5.280052)	8.864413 (±6.943758)
Best fitness		1.504085	1.550623	1.425379	1.377808

*The fitness is the total reaching distance, in meters, on the testing set, thus the smaller the better*.

*N.A., not applicable*.

The star (*) indicates values that reached their maximum or minimum allowed value.

Table 2 presents the best hyperparameters for all tested conditions and the corresponding fitness value. The hyperparameter values obtained for most parameters are well within the ranges defined in Table 1. The only exception were for CACLA + var beta in the *reward only* and *reward* + *nociception* conditions, and the learning rate for the Critic in *reward only* condition.

### 3.1 Effect on Positioning Error

**Figure 4:**
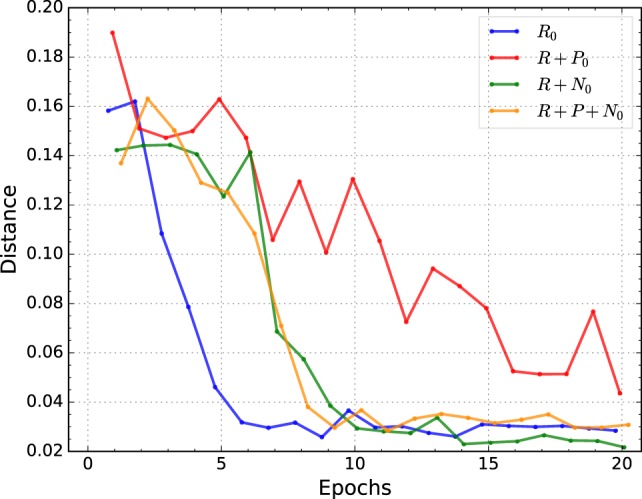
**Mean positioning error of the best individuals of each condition**. All conditions but *reward* + *punishment* converge fast and reach small positioning error in a small number of epochs.

Figure 4 shows the average performance of the best individual for each condition over 20 epochs. The average is obtained by training 10 randomly initialized networks with the best-evolved hyperparameters of the corresponding conditions and tested on the validation set.

All conditions but *reward* + *punishment* have a similar convergence pattern, i.e., they reduce the positioning error sharply during the first 10 epochs and it is further reduced thereafter but at a much lower rate. The *reward* + *nociception* condition consistently reaches a lower mean value than all other conditions. Additionally, in contrast to all other conditions, *reward* + *nociception* shows a much smaller oscillatory behavior after convergence. Also interesting is the fact that the *reward* + *punishment* condition shows a slower convergence speed.

A two-way ANOVA of the average performance on the validation set of the best 4 individuals for each condition tested with 10 different random initializations provides additional evidence for a difference between the tested conditions. Post-processing this ANOVA with a pairwise comparison (“Tukey-Kramer”), the difference between conditions is narrowed down to *reward only*, our baseline, and the *reward* + *nociception* condition, see Table 3. The use of nociceptive units leads to improvements in the total positioning error when compared to agents trained in the *reward only* condition while the use of punishment seems to have no effect on performance.

**Table 3:** **Pairwise comparison for the average positioning error and convergence speed using “Tukey-Kramer” for a 95% confidence interval on a two-way ANOVA**.

	Group 1	Group 2	Lower	Mean diff.	Upper	p-Value	Mean diff (%)
Performance	*R*	*R* + *P*	−0.0121	0.0028	0.0177	0.9627	7.18
	*R*	*R* + *N*	−0.0027	0.0122	0.0271	0.1523	31.28
	*R*	*R* + *P* + *N*	−0.0114	0.0036	0.0185	0.9278	9.23
	*R* + *P*	*R* + *N*	−0.0055	0.0094	0.0243	0.3681	25.97
	*R* + *P*	*R* + *P* + *N*	−0.0142	0.0008	0.0157	0.9992	2.21
	*R* + *N*	*R* + *P* + *N*	−0.0236	−0.0086	0.0063	0.4442	−32.09
Speed	*R*	*R* + *P*	−0.7232	−0.5180	−0.3129	0.0000	−44.29
	*R*	*R* + *N*	−0.1879	0.0173	0.2224	0.9964	1.48
	*R*	*R* + *P* + *N*	−0.4286	−0.2234	−0.0183	0.0264	−19.10
	*R* + *P*	*R* + *N*	0.3302	0.5353	0.7405	0.0000	31.72
	*R* + *P*	*R* + *P* + *N*	0.0894	0.2946	0.4997	0.0013	17.46
	*R* + *N*	*R* + *P* + *N*	−0.4459	−0.2407	−0.0356	0.0137	−20.89

*Here, we analyze the average performance and convergence speed on the validation set of the best 4 hyperparameter sets for each condition. All hyperparameter sets were tested with 10 different randomly initialized networks. A positive percentage indicates the effect size in which Group 2 improves upon Group 1, whereas a negative percentage indicates the effect size in which Group 2 is worse than Group 1*.

The same analysis on the convergence speed, measured as the area under the curve for all 20 epochs, is more categorical, see Table 3. Here, both conditions trained with punishment significantly reduce the convergence speed compared to our baseline. On the other hand, the use of nociceptive units partially counteracts the negative effect of punishment, and when used without punishment nociception performs as well as our baseline.

### 3.2 Effect on the Perceived Nociception

**Figure 5:**
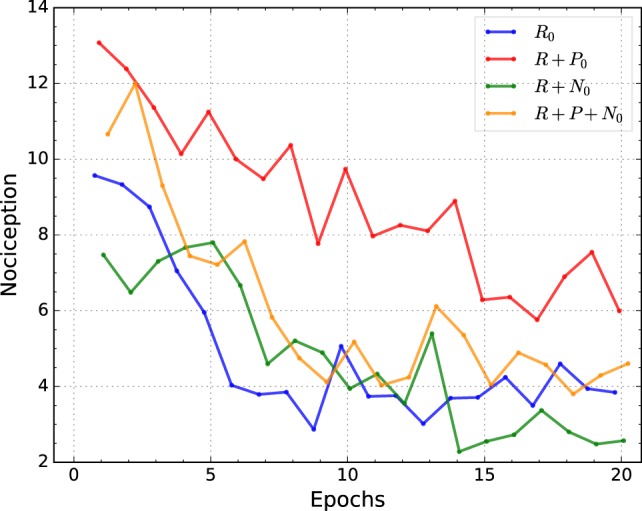
**Average measured nociception for both joints, for all samples in the validation set and for the best individuals of each condition**. All four conditions show an oscillatory convergent behavior. The condition trained with reward and nociceptive units converges to a smaller value than all other conditions.

Figure 5 shows the perceived nociception (potential for damage) of the best individuals for each condition over 20 epochs. The average is obtained by training 10 randomly initialized networks with the best-evolved hyperparameters of the corresponding conditions and tested on the validation set. The mean perceived nociception or *potential for damage* is computed based on the cumulative absolute value of nociception per joint, as defined in equation (7), and per time step for all samples on the validation set after learning. The maximum number of steps is limited to 10. Thus, the maximum perceived nociception per sample in the validation set is 20.

Nociception was measured in all conditions; however, it is only used as input for the *reward* + *nociception*, and *reward* + *punishment* + *nociception* conditions. Nociception and punishment were only used for learning and have no influence on the hyperparameter optimization. Nevertheless, the *reward* + *nociception* condition consistently reduce the potential for damage as shown in Figure 5. Similarly, as for the results of positioning error, the larger reduction on measured nociception occurs before the 10th epoch, after which the reduction is smaller and an oscillatory behavior for all conditions is observed.

When observing the average *potential for damage* after learning, the use of nociceptive units seems to reduce the total potential for damage. However, unlike for positioning error, the reduction of nociception is not as large (% difference) or reliable (p-value), see Table 4. By contrast, punishment seems to have a negative effect on the potential for damage when compared to our baseline and a significant negative effect when compared to the *reward* + *nociception* condition.

**Table 4:** **Pairwise comparison for nociception using “Tukey-Kramer” for a 95% confidence interval on a two-way ANOVA**.

	Group 1	Group 2	Lower	Mean diff.	Upper	p-Value	Mean diff (%)
After learning	*R*	*R* + *P*	−2.4641	−0.9040	0.6560	0.4443	−22.90
	*R*	*R* + *N*	−0.6973	0.8627	2.4228	0.4863	21.85
	*R*	*R* + *P* + *N*	−2.2731	−0.7131	0.8470	0.6431	−18.07
	*R* + *P*	*R* + *N*	0.2068	1.7668	3.3268	0.0190	36.42
	*R* + *P*	*R* + *P* + *N*	−1.3691	0.1910	1.7510	0.9892	3.93
	*R* + *N*	*R* + *P* + *N*	−3.1358	−1.5758	−0.0158	0.0466	−51.08
During learning	*R*	*R* + *P*	−58.5381	−47.5636	−36.5891	0.0000	−49.86
	*R*	*R* + *N*	−8.0196	2.9549	13.9294	0.9003	3.10
	*R*	*R* + *P* + *N*	−36.5662	−25.5917	−14.6172	0.0000	−26.82
	*R* + *P*	*R* + *N*	39.5440	50.5185	61.4930	0.0000	35.34
	*R* + *P*	*R* + *P* + *N*	10.9973	21.9718	32.9463	0.0000	15.37
	*R* + *N*	*R* + *P* + *N*	−39.5212	−28.5466	−17.5721	0.0000	−30.88

*Here, we analyze the average perceived nociception on the validation set of the best 4 hyperparameter sets for each condition, both after learning as well as the cumulative absolute value during learning. All hyperparameter sets were tested with 10 different randomly initialized networks. A positive percentage indicates the effect size in which Group 2 improves upon Group 1, whereas a negative percentage indicates the effect size in which Group 2 is worse than Group 1*.

Again, if we consider the average cumulative absolute value of perceived nociception during learning, punishment significantly increase the potential for damage by almost 50%. Similarly, as for the positioning error, nociception units can partially counteract the negative effect of punishment, while performing similarly to our baseline.

### 3.3 Effect on Positioning Speed

**Figure 6:**
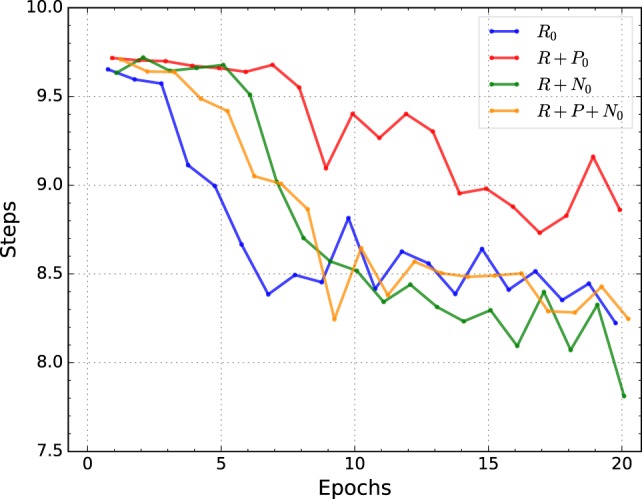
**Average number of steps needed to position the robot’s arm for the best individuals of each condition**.

Figure 6 shows the average positioning speed of the best individuals for each condition over 20 epochs. The average is obtained by training 10 randomly initialized networks with the best-evolved hyperparameters of the corresponding conditions and tested on the validation set.

Figure 6 shows that the condition trained with nociceptive units effectively and consistently reduce the number of steps needed to position the robot’s arm, even though the hyperparameter optimization procedure only attempted to reduce the total positioning error, see Table 5. Similarly, as for the previous two metrics, the gains on positioning speed are larger until the 10th epoch. In contrast to the previous metrics, here all conditions show a marked oscillatory behavior, see Figure 6.

**Table 5:** **Pairwise comparison for positioning speed using “Tukey-Kramer” for a 95% confidence interval on a two-way ANOVA**.

	Group 1	Group 2	Lower	Mean diff.	Upper	p-Value	Mean diff (%)
After learning	*R*	*R* + *P*	−0.5872	−0.2549	0.0774	0.1992	−3.04
	*R*	*R* + *N*	−0.0417	0.2906	0.6230	0.1109	3.47
	*R*	*R* + *P* + *N*	−0.3855	−0.0532	0.2791	0.9765	−0.63
	*R* + *P*	*R* + *N*	0.2132	0.5455	0.8779	0.0001	6.31
	*R* + *P*	*R* + *P* + *N*	−0.1306	0.2017	0.5340	0.4022	2.34
	*R* + *N*	*R* + *P* + *N*	−0.6762	−0.3438	−0.0115	0.0393	−4.25
During learning	*R*	*R* + *P*	−8.2838	−6.0490	−3.8143	0.0000	−3.45
	*R*	*R* + *N*	0.3106	2.5453	4.7801	0.0180	1.45
	*R*	*R* + *P* + *N*	−4.0134	−1.7786	0.4561	0.1717	−1.01
	*R* + *P*	*R* + *N*	6.3596	8.5944	10.8291	0.0000	4.74
	*R* + *P*	*R* + *P* + *N*	2.0356	4.2704	6.5051	0.0000	2.35
	*R* + *N*	*R* + *P* + *N*	−6.5587	−4.3240	−2.0892	0.0000	−2.50

*Here, we analyze the average positioning speed and the cumulative number of steps needed during learning on the validation set of the best 4 hyperparameter sets for each condition. All hyperparameter sets were tested with 10 different randomly initialized networks. A positive percentage indicates the effect size in which Group 2 improves upon Group 1, whereas a negative percentage indicates the effect size in which Group 2 is worse than Group 1*.

Overall, the effect size for the positioning speed is small for both, after learning and during learning. When observing the positioning speed after learning and similarly as for the potential for damage, here nociceptive units also seem to improve performance. Although the effect is more reliable than the one seen in the reduction of the potential for damage, the actual effect is smaller. By contrast and similar to what was seen for the potential for damage, punishment seems to have a detrimental effect on the positioning speed when compared to the baseline condition (*reward only*). Punishment is significantly detrimental when compared to the best performing condition on this metric, i.e., *reward* + *nociception*.

When observing the cumulative effect, nociception units once again counteract the negative effect of punishment. However, for this metric, nociceptive units perform significantly better than our baseline but the effect size is rather small.

## 4 Discussion

The results shown in Section 3 indicate that the use of nociceptive units can improve learning performance and behavioral performance. This improvement is observable across different evaluation metrics, i.e., positioning error, the potential for damage, and positioning speed. This result is noteworthy if we consider that the algorithms were only optimized to reduce positioning error.

A more detailed comparison among the best solutions for each condition shows that the metrics with a more substantial improvement are positioning error and potential for damage, when compared to conditions trained with *reward only*, see Section 3.1 and Section 3.2. Our results also show that the largest improvement in all three metrics is obtained when using nociceptive units only. We believe that by enlarging the state space with the nociceptive units we are effectively projecting the input vector into a higher dimensional space, thus magnifying the differences between input vectors. This is analogous to the phenomenon observed in reservoir computing, where input vectors are transformed to a dynamic higher dimensional space thus reducing ambiguity between similar input vectors. By contrast, punishment seems to have a negative impact on all three metrics when compared to our baseline, the *reward only* condition. The negative effect of punishment is even larger when the conditions trained with punishment are compared to the *reward* + *nociception* condition. The negative impact of punishment could also be inferred from the results of the hyperparameter optimization. Here we can see that the best value for punishment converges to the smallest punishment possible, in this case, −0.1.

We believe that the conditions that use punishment have a more complex solution space, this can be observed with respect to the speed of convergence for all three metrics evaluated here. This could be supported by the twice as large mean fitness scores of the conditions trained with punishment when compared to the *reward* + *nocipcetion* condition, see Table 2. Computationally, we hypothesize that the oversimplification of punishment, i.e., being treated as a negative reward and being conflated with reward as a single scalar value, cf. *r_t_* in equation (1), creates ambiguity and thus makes the problem harder. For instance, consider the case where a high reward of 100 co-occurs with a high punishment of −90: the resulting reward value fed into *r_t_* in equation (1) would be 10 which is indistinguishable from a case where only a small reward of 10 is present and no punishment.

For biological systems, Dayan and Niv (2008) argued that the heterogeneous range of effects that aversive predictions elicit, which greatly depend on contextual information, may be the reason for it. This would be in agreement with existing evidence that indicates that punishment-driven learning may be a more complex and cognitively demanding process than reward-driven learning (Palminteri and Pessiglione, 2017). Specifically, during feedback receipt, punishment-driven, in comparison to reward-driven, learning elicits a greater engagement of the prefrontal cortex and thus more attentional and cognitive resources are recruited (Kim et al., 2015).

Furthermore, evidence suggests that there are substantial neurobiological differences between reward- and punishment-driven learning (Wächter et al., 2009; Kim et al., 2015). For instance, the striatum, the amygdala, and the medial OFC seem to be more involved in reward-driven learning, while for punishment-driven learning, the insula or the lateral OFC play a greater role (Wächter et al., 2009; Kim et al., 2015).

We presented nociceptive units as a simple way of enhancing the performance of neurally implemented TD-learning algorithms *by increasing the state space*. We showed that the use of nociceptive units can improve the overall learned behavior, i.e., reduce the positioning error, reduce the potential for damage, and increase the positioning speed. Alternatively, we showed that punishment signals may be detrimental to the overall learned behavior. This may be caused by the loss of predictive power of conventional TD-learning algorithms as pointed out by Lowe and Ziemke (2013).

The results above shed light on the, to some extent, neglected differential effects of reward- and punishment-driven learning in the reinforcement learning literature. Moreover, for certain tasks such as inverse kinematic learning, nociceptive units seem to carry an unexpected boost in performance. Thus, we urge further study of the effect of punishment-driven learning, the combined effect of both types of reinforcer on behavior learning, and the higher dimensional sensorimotor input in order to refine biological models of learning by reinforcement with the added benefit of potentially improving the computational effectiveness of TD-learning methods, as shown in this work.

As future work, we aim to study the underlying mechanism(s) that lead to the improvements caused by the use of nociceptive units. Once identified, it would be possible to conceptualize procedures to further increase these beneficial effects or envisage ways to apply the concept of nociception to non-embodied reinforcement learning problems. This could even lead to a universal approach of extending TD-learning algorithms, which could resemble the feedback provided by nociceptor distributed throughout the body.

Other more specific extensions would be to study the effect of punishment and nociceptive units on robot pose or to further develop our current implementation to learn the inverse kinematics of a real humanoid robot arm.

## Author Contributions

NN-G designed and performed the experiments. All authors contributed to the interpretation and structuring of the results, and wrote the paper.

## Conflict of Interest Statement

The authors declare that the research was conducted in the absence of any commercial or financial relationships that could be construed as a potential conflict of interest.

## Footnotes

^1^https://www.iasp-pain.org/.

^2^http://doc.aldebaran.com/2-1/family/nao_h25/links_h25.html.

^3^http://doc.aldebaran.com/2-1/family/nao_h25/joints_h25.html.
